# COVID-19 and Tuberculosis: Mathematical Modeling of Infection Spread Taking into Account Reduced Screening

**DOI:** 10.3390/diagnostics14070698

**Published:** 2024-03-26

**Authors:** Anna Starshinova, Nikolay Osipov, Irina Dovgalyk, Anastasia Kulpina, Ekaterina Belyaeva, Dmitry Kudlay

**Affiliations:** 1 Almazov National Medical Research Centre, 197341 St. Petersburg, Russia; asya.starshinova@mail.ru; 2 Department of Steklov Mathematical, Institute of Russian Academy of Sciences, 191023 St. Petersburg, Russia; nicknick@pdmi.ras.ru; 3 Mathematical Department, St. Petersburg State University, 199034 St. Petersburg, Russia; 4 Research Institute of Phthisiopulmonology, 190961 St. Petersburg, Russia; prdovgaluk@mail.ru; 5 Medical Department, State Pediatric Medical University, 194100 St. Petersburg, Russia; 6 Republic TB Healthcare Dispensary, 185032 Petrozavodsk, Russia; ekaterina_83@bk.ru; 7 Department of Pharmacology, I.M. Sechenov First Moscow State Medical University, 119435 Moscow, Russia; d624254@gmail.com; 8 Immunology Department, Institute of Immunology FMBA, 115552 Moscow, Russia

**Keywords:** tuberculosis, COVID-19, screening for tuberculosis, incidence, bacterial excretion, mathematical model, dynamic system

## Abstract

The COVID-19 pandemic resulted in the cessation of many tuberculosis (TB) support programs and reduced screening coverage for TB worldwide. We propose a model that demonstrates, among other things, how undetected cases of TB affect the number of future *M. tuberculosis* (*M. tb*) infections. The analysis of official statistics on the incidence of TB, preventive examination coverage of the population, and the number of patients with bacterial excretion of *M. tb* in the Russian Federation from 2008 to 2021 is carried out. The desired model can be obtained due to the fluctuation of these indicators in 2020, when the COVID-19 pandemic caused a dramatic reduction in TB interventions. Statistical analysis is carried out using R v.4.2.1. The resulting model describes the dependence of the detected incidence and prevalence of TB with bacterial excretion in the current year on the prevalence of TB with bacterial excretion in the previous year and on the coverage of preventive examinations in the current and previous years. The adjusted coefficient of model determination (adjusted R-squared) is 0.9969, indicating that the model contains almost no random component. It clearly shows that TB cases missed due to low screening coverage and left uncontrolled will lead to a significant increase in the number of new infections in the future. We may conclude that the obtained results clearly demonstrate the need for mass screening of the population in the context of the spread of TB infection, which makes it possible to timely identify patients with TB with bacterial excretion.

## 1. Introduction

Tuberculosis (TB) still remains a deadly infectious disease that leads to a large number of deaths worldwide [1,2]. Before the COVID-19 pandemic, a quarter of the world’s population was infected by *M. tuberculosis* (*M. tb*), according to the estimation of the WHO. However, most people did not develop active TB [3,4]. An approximate estimation showed that about 90% of people infected by *M. tb* are adults [5,6].

The appointment of correct and timely treatment with the use of basic antituberculosis drugs makes it possible to cope with the infection and achieve a cure in 85% of cases with preserved pathogen sensitivity to drugs and about 75% in cases of drug-resistant *M. tb* [2,7,8].

The novel COVID-19 infection had a significant impact on the well-established processes of detection and support programs for TB patients in many countries around the world [9,10,11]. The WHO data obtained in 2021 demonstrated a clear trend towards a decrease in newly detected cases of the disease in all countries of the world, including countries with a high burden of the disease [2]. At the same time, the analysis [12,13] showed that skipping pathology is expected in 40% of cases in children and in 21% of cases in adults.

Starting from the first months of the COVID-19 pandemic, scientists began to model the further epidemic situation regarding morbidity and mortality from TB and HIV infection, identifying important factors influencing the aggravation of the situation in the future [3,14,15,16].

The Russian Federation is one of the few countries in the world where mass screening of TB infection is carried out both among adults and among children [17,18,19,20]. X-ray examination is the main method of screening for TB infection among the adult population and adolescents in the country. It makes it possible to detect changes in the lungs for further additional examination in TB facilities [21,22]. Children under 15 years old are examined using the Mantoux test with 2 TU from 1 to 8 years old, and then up to 15 years old, immunodiagnostics is carried out with a new Russian skin test: a recombinant tuberculosis allergen (test with Diaskintest^®^ (Generium, Moscow, Russia)) [17,23,24]. In 2022, the Diaskintest^®^ was recommended by the WHO [25].

In the presence of a fairly well-established system for detecting TB patients at the screening stage, it must be recognized that during the COVID-19 pandemic, there were certain objective difficulties in applying well-established processes for preventive examination for TB [16,26]. Understanding the risks associated with reduced coverage of mass screening for TB is an important task. Carrying out mathematical analysis and modeling will allow us to achieve the goal.

## 2. Materials and Methods

This section contains a preliminary description of the data and how we intend to work with them, as well as a brief listing of statistical tools we intend to use further in Section 3, where our main considerations and results will be presented.

### 2.1. Data and Study Design

This study relies on data from the official statistics of the Russian Federation on TB infection [27]. The official statistics data used for modeling are three time series for the period from 2008 to 2021. The analysis of the following main parameters is carried out, the significance of which and the correlation with the epidemic situation were proved earlier [16]: coverage with preventive examinations for TB (in percent), incidence of TB (number of newly diagnosed cases per 100,000 population), and the prevalence of patients with bacterial excretion registered at the end of the year (total number of patients with *M. tb* per 100,000 population) [28,29]. These data are presented in Table 1.

What draws attention is the similarity of the detected incidence rate in the current year with the bacillarity level of last year, as well as the sharp violation of this similarity in 2020 amid a sharp decrease in the percentage of the population screened for TB.

At the first stage, we conducted a preliminary statistical analysis confirming that the incidence drop in 2020 was indeed anomalous. The analysis carried out leads us to form the following hypotheses:

- The drop in TB screening has resulted in the under-detection of TB cases.
- Measures aimed at combating the pandemic also led to a sharp decrease in the incidence of TB. However, such an alternative interpretation directly contradicts the trends that were observed worldwide, as reflected in the WHO report [2].
- All TB cases missed due to a decrease in TB screening significantly affected the incidence and prevalence of TB with bacterial excretion, namely led to the growth of the number of epidemic foci inside the country.

We build a simple mathematical model that describes the dependence of the incidence of TB and the prevalence of TB with bacterial excretion in the current year on the prevalence of TB with bacterial excretion in the previous year and on the screening coverage in the current and previous years. The construction of this model became possible only due to the unusual change in indicators in 2020 during the COVID-19 pandemic. The almost deterministic model is a dynamic system with control, where control is preventive examination coverage. We use it to show that missed due to low TB screening coverage and remaining undiagnosed cases will lead to a significant increase in the number of TB patients in the future due to the increase in the “bacillary core” in the country. Namely, we quantify this growth by modeling and comparing two scenarios for the next five years, with low and high coverage of preventive examinations, respectively.

### 2.2. Statistical Methods

All calculations are performed using R v.4.2.1 [30]. All data are normalized to the logit scale. The hypothesis that the drop in detected incidence in 2020 was anomalous is tested by the Grubbs test [31], which is implemented in package outliers [32] and designed to search for outliers in data. We also apply the Ljung–Box test [33] and the Shapiro–Wilk test [34] to test the independence and normality of the remaining annual changes in incidence. After bringing the data to the logit scale, all dependencies in the models below become linear, and standard linear regression analysis is used to find appropriate coefficients. Using it, we derive the above-mentioned dependence of the incidence of TB and the prevalence of TB with bacterial excretion in the current year on the prevalence of TB with bacterial excretion in the previous year and on the screening coverage in the current and previous years.

## 3. Models and Results

### 3.1. Preliminary Analysis

We normalize the data to the logit scale, where linear transformations and hypotheses of normality become formally admissible. By *S_t_*, *I_t_*, and *M_t_*, we denote the time series of initial indicators: examination coverage, incidence, and prevalence of TB patients with bacterial excretion, respectively. We denote the transformed time series as

*s_t_* = logit (*S_t_*/100), *i_t_* = logit (*I_t_*/100,000), and *m_t_* = logit (*M_t_*/100,000).

For illustration, the resulting values are shown in Table 2.

The aforementioned similarity between the detected incidence in the current year and the prevalence of TB with bacterial excretion in the previous year, as well as the absence of this pattern in 2020, has become more apparent.

Next, based only on the incidence data, it may be noted that there was an abnormally sharp decrease in the number of newly diagnosed cases in 2020 (Table 3).

**Figure 1:**
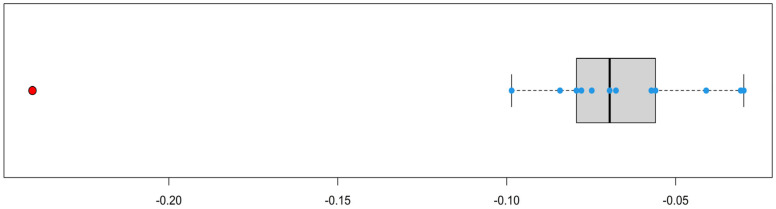
Visualization of the sample *i_t_ − i_t−_*_1_*.* The blue dots are normal differences and the red dot is an outlier.

To confirm the statistical significance of this fact, we make sure that the time series *i_t_* − *i*_*t*−1_ of first differences looks like a sample generated by mutually independent, identically and normally distributed random variables with an outlier at the point corresponding to the difference between 2019 and 2020. Indeed, applying the Ljung–Box test for independence [33] and the Shapiro–Wilk test for normality [34] to the considered sequence of differences without the abnormal point, we achieve *p*-values equal to 0.590 and 0.605, respectively. Next, we apply, to the entire sample of differences, the boxplot function, which finds outliers using the interquartile range and builds a box plot. The result is shown in Figure 1, where the red dot is the outlier that corresponds to the difference between 2019 and 2020.

Also, the normality of the studied sample allows us to apply a more subtle Grubbs test [31], implemented in the package outliers [32] and designed to search for outliers; the *p*-value for the hypothesis that the discussed difference *i*_2020_ − *i*_2019_ is not an outlier turns out to be equal to 4.97 × 10^−5^, which leads us to the alternative hypothesis that it is an outlier. In other words, in 2020, there was a decrease in the detected incidence that was, under the influence of certain factors, abnormally sharp with high statistical significance.

At the same time, just as in 2020, there was a drop in TB screening coverage due to the COVID-19 pandemic, the only time in the considered time interval between 2008 and 2021.

### 3.2. Incidence Model

Looking at Table 2, we can assume that the incidence in the current year depends mainly on the number of TB patients with bacterial excretion in the previous year. Such patients form the “bacillary core” in the population. At the same time, the indicators obtained during the pandemic suggest that there are also second-order effects: reduced screening coverage leads to missed TB cases and a lower incidence rate in the same year. These missed TB cases may be detected in the next year, which is expected to increase the corresponding incidence rate. Thus, we can expect the model to look as follows:

*i_t_* = *β*_0_ + *β*_1_
*m*_*t*−1_ + *β*_2_
*s*_*t*−1_ + *β*_3_
*s_t_* + *ε_t_*,

where *β*_1_ is close to 1, *β*_2_ < 0, and *β*_3_ > 0. Indeed, the regression analysis leads us to the results presented in Table 4.

Based on the results of the analysis, the following conclusions can be drawn.

- The obtained *p*-values for the hypotheses of insignificance of the coefficients are small: the statistical significance of the dependence on all three parameters is very high.
- The coefficient values are as expected. In particular, the signs at the coefficients *β*_2_ and *β*_3_ indicate that high screening coverage increases the current number of detected cases and reduces their number in the future.
- The general indicators presented at the end of Table 4 show that the model is highly accurate and contains almost no probabilistic component.
- It can be seen that without data corresponding to time point *t* = 2020 (the COVID-19 pandemic), nothing but the relationship between the incidence of TB and the prevalence of TB with bacterial excretion would have been possible to establish.

### 3.3. Model of Prevalence of TB with Bacterial Excretion

The incidence model obtained above does not directly say that cases missed due to low screening coverage will lead to an increase in the number of infections. So far, we have only reliably seen that missed cases being detected in the future will increase the incidence rate. However, the fact that the absolute value of *β*_2_ significantly exceeds *β*_3_ may indirectly indicate that those patients who are left without control can infect a larger number of people. We will show that this *third-order effect* (by the first-order effect, we mean that each patient with bacterial excretion produces approximately one new case in the next year, and the secondary effect is that cases missed in the current year may be detected in the next year, resulting in an increase in the corresponding incidence rate) really takes place by constructing two scenarios with low and high coverage of preventive examinations, respectively.

In order to build such scenarios, we also need another model that predicts bacterial excretion. It is clear that the number of patients with bacterial excretion, registered by the end of the year, depends on the number of such patients in the past year and on the current incidence. As we already know, the latter also depends on last year’s prevalence of patients with bacterial excretion, as well as on current and last year’s coverage of examinations. This brings us to a model similar to the one we have used for incidence:

*m_t_* = *γ*_0_ + *γ*_1_
*m*_*t*−1_ + *γ*_2_
*s*_*t*−1_ + *γ*_3_
*s_t_* + *ε_t_*.

The results of the regression analysis for this model are presented in Table 5.

Here, we can make the same conclusions as those listed after Table 4.

### 3.4. Implications of the Models

**Figure 2:**
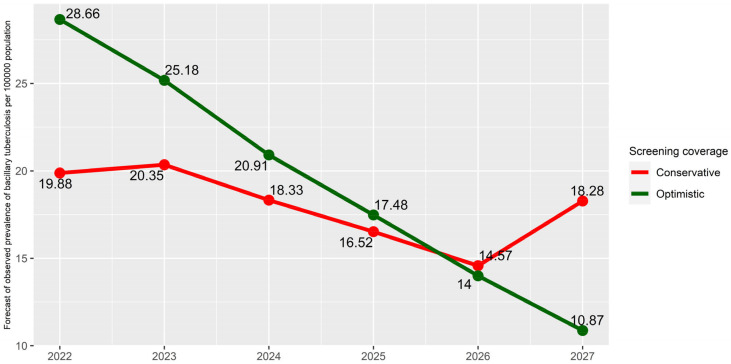
Forecast of prevalence of TB with bacterial excretion.

**Figure 3:**
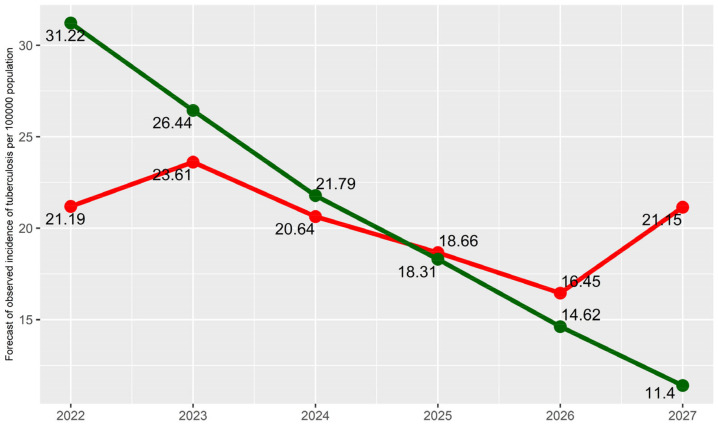
TB incidence forecast.

In the next step, we build the two scenarios that we have talked about earlier: low and high coverage of preventive examinations, respectively. For these scenarios, we use the values of population screening coverage that were in the conservative and optimistic scenarios in [16]. We also add an extra time point at the end with the same value of screening coverage in 90% for both scenarios. Using the two models obtained above and moving from the logit scale to the original one, we obtain the results presented in Table 6 and Table 7. The patterns obtained are also presented in Figure 2 and Figure 3.

In Table 6 and Figure 2, we can see that at first, higher screening coverage would lead to better detection and a greater number of registered patients with *M. tb*, but by 2026, there would be fewer of them than with lower coverage, even without taking into account those patients who would not have been identified in the conservative scenario. Moreover, when in 2027, in both scenarios, examinations are assumed to be conducted in 90% of the population, and in the conservative scenario, undiagnosed patients are also detected, there would be 1.68 times more of them than in the optimistic scenario.

In Table 7 and Figure 3, we see a similar pattern for TB incidence. First, the incidence would be higher in the optimistic scenario due to better detection of TB patients, but from 2025, the situation would be reversed. Finally, in 2027, previously undetected TB patients would be identified, and the incidence in the conservative scenario would become 1.86 times higher than in the optimistic one (Table 8).

We had an opportunity to compare the values predicted by the models with the real epidemiological data of 2022. The resulting models demonstrate correctness, interpretability, and consistency with each other. However, as we can see, the predicted values of the 2022 indicators are slightly lower than the real ones. Based on these two facts, we can assume that in 2022, a certain external factor caused a worsening of the epidemiological situation with TB.

We can confirm the impact of the mentioned factor in another way. Namely, by adding a new point *i*_2022_ − *i*_2021_ = 0 to the sample from Table 3 and applying a variant of the Grubbs test for two outliers on opposite tails, we obtain a *p*-value equal to 0.0085. This supports the hypothesis that both points *i*_2020_ − *i*_2019_ and *i*_2022_ − *i*_2021_ are outliers, and there is a certain external reason why the TB incidence did not decrease in 2022.

## 4. Discussion

Screening and early detection of patients, using both immunological and radiological methods, are the main measures for controlling the spread of TB infection [18,35,36,37]. However, already in 2021, WHO stated that about 4.1 million TB patients in 2020 were not diagnosed with TB or were not included in the official statistics of countries [2].

Thanks to the well-established system of preventive examination for TB in the Russian Federation, it was possible to cope with the severe epidemic situation that emerged after the collapse of the Soviet Union [18]. As we can see in Table 1, from 2009 to 2021, there was a systematic decrease in the incidence of TB.

It is obvious that the screening of TB infections is most effective with the use of immunologic methods when we need to diagnoses a latent TB infection. These measures are possible to carry out preventive treatment in people with a high risk of active TB [5,20,24,37].

It should be noted that immunological methods are most often used in high-risk groups in world practice [5,38]. According to many studies, the high informative value of in vitro tests (ELISPOT (Oxford Immunotec Ltd., Abingdon, UK), QuantiFERON-TB Gold, and QuantiFERON-TB Gold Plus (Qiagen, Hilden, Germany) in the early diagnosis of TB infections in both adults and children has been proven [39,40,41,42]. However, we should take into account the high cost and the need to organize highly qualified laboratory diagnoses for these tests [43].

At the same time, ELISPOT remains the method of choice in people with immunosuppression, associated with the ability of the test to determine the activity of TB infection even in patients with HIV infection with severe immunosuppression [39,44].

Skin tests have historically been widely used since the time of Robert Koch because they are simple to use, low-cost, and highly informative [45,46]. Today, while there is no doubt about the high sensitivity of the Mantoux test with 2 TU, in the context of vaccination against TB, the test has a low specificity [47,48]. The development and application of a new skin test in the Russian Federation, the Diaskintest^®^, address this problem and make it possible to screen for TB infection in the context of vaccine prophylaxis with high information content [48,49].

At the same time, all the skin tests have certain limitations for TB in patients with immunosuppression. These tests show less effectiveness in comparison with IGRA tests [23,44]. In this connection, the search for new or additional biomarkers for TB diagnosis with greater information content continues [50,51].

At the same time, X-ray methods are much cheaper, make TB screening possible without significant difficulties, such as creating additional laboratories, and can be applied in remote regions of the country [16,22,52].

Due to disruptions in the work of the well-established program for preventive examination of the population of the Russian Federation during COVID-19, it is possible to obtain adverse long-term results that affect the change in the epidemic situation of TB [15,26].

In our mathematical analysis, we have shown that a decrease in TB screening coverage will lead to an increase in the number of uncontrolled cases of TB with bacterial excretion and, as a consequence, to an increase in the rate of spread of TB. With a high prevalence of HIV infection in the country, the growth of the “bacillary core” will immediately lead to an increase in mortality from TB in combination with HIV infection. This scenario will require additional resources and interventions to prevent the spread of infection within a country or region [53].

Under these conditions, the creation of regional databases and personal monitoring of the diagnosis and effectiveness of treatment for patients with TB can be a real tool for correcting the current situation. Today, specialists in radiologic diagnostics talk about the need for more active use of artificial intelligence and combining the efforts of all communities to analyze data in order to detect TB in a timely manner, even at the stage of the appearance of changes in the lungs [54]. Some researchers draw attention to the need for wider use of MRI research, especially for the detection and diagnosis of extrapulmonary TB [55,56]. However, under the conditions of mass screening, the MRI study can be complicated by the high cost of the method [57,58,59]. Today, with the expansion of the use of online technologies, telemedicine has become a new method for identifying, analyzing, and monitoring in the management of patients with various pathological conditions, including TB [60,61].

At the same time, in the absence of adequate and enhanced measures for the early detection of TB, an increase in mortality from TB can be expected, especially in countries with a high burden of TB infection. It’s necessary to take into account the change in the immunological status of patients who have undergone COVID-19, namely immunosuppression [62,63]. The development of an immunosuppressive state will influence the results of skin tests and the development of the TB process [49]. In general, the use of immunological tests will allow early diagnosis of TB infection for timely preventive treatment after the exclusion of active TB. At the same time, X-ray examination can be an additional and cheap method of examination to detect lung changes for additional examination. In the present conditions, it is necessary to take into account the possibility of using telemedicine with remote consultation and analysis of the data obtained during the examination. The COVID-19 pandemic had a significant impact on the detection of TB patients in all countries of the world, which was associated with the objective situation of the spread of SARS-CoV-2 virus in 2020–2021.

It is also worth noting that the fact that undetected cases have a very serious impact on the rate of spread of an infectious disease is presented in the literature (see, e.g., [64,65] for COVID-19), including among migrants [66].

## 5. Conclusions

The main contribution of the study is an understanding of the risks associated with reduced coverage of mass screening for TB. The resulting models have a clear interpretation, high accuracy, and almost no probabilistic component. Furthermore, all dependencies have very high statistical significance. The models imply that a decrease in TB screening coverage will lead to an increase in the number of uncontrolled cases of TB with bacterial excretion and, as a consequence, to an increase in the rate of spread of TB. According to our data, the predicted values spread TB with bacterial excretion of the 2022 indicators are slightly lower than the real ones (25.6% vs. 26.6%). Under present conditions, according to the data we have obtained, an increase in the detected TB incidence can be expected if all interventions to identify patients are carried out correctly and in a timely manner.

In the absence of adequate and intensified early detection of TB, an increase in TB mortality can be expected, especially in countries with a high burden of TB infection. The number of bacteriologically isolated tuberculosis patients reported by the end of the current year should depend on the number of such patients in the previous year and the current incidence in the country. These data will be influenced by the number of people X-ray examined the year before.

It should be noted that our main result is an accurate and interpretable model of the dependency between mass TB screening and TB infection spread. It shows how TB cases missed due to low screening coverage and left uncontrolled lead to a significant increase in the number of new infections in the future. The role of COVID-19 for our study is important, but rather technical: the construction of the model became possible due to the fluctuation of TB indicators in 2020 during the COVID-19 pandemic.

## Figures and Tables

**Table 1:** Dynamics of epidemiological data.

Year	2008	2009	2010	2011	2012	2013	2014	2015	2016	2017	2018	2019	2020	2021
Population screening for TB (%)	61.4	62.5	63.8	64.4	65.7	65.8	66.6	68.1	69.3	71.3	72.7	73.7	66.7	71.0
Incidence of TB (per 100,000)	85.1	82.6	77.2	73.0	68.1	63.0	59.5	57.7	53.3	48.3	44.4	41.2	32.4	31.1
The prevalence of TB with bacterial excretion (per 100,000)	79.5	77.2	72.9	69.0	64.7	60.5	56.8	54.0	50.7	46.0	42.8	37.8	30.7	28.5

The color scheme in the table emphasizes the similarity (diagonally) between the current incidence and last year’s bacillarity.

**Table 2:** Epidemiological indicators in the logit scale.

*T*	2008	2009	2010	2011	2012	2013	2014	2015	2016	2017	2018	2019	2020	2021
*s_t_*	0.46	0.51	0.57	0.59	0.65	0.65	0.69	0.76	0.81	0.91	0.98	1.03	0.69	0.89
*i_t_*	−7.07	−7.10	−7.17	−7.22	−7.22	−7.37	−7.43	−7.46	−7.54	−7.64	−7.72	−7.79	−8.03	−8.08
*m_t_*	−7.14	−7.17	−7.22	−7.28	−7.34	−7.41	−7.47	−7.52	−7.59	−7.68	−7.76	−7.88	−8.09	−8.16

Here *t* stands for year, and *s_t_*, *i_t_*, *m_t_* stand for screening coverage, incidence and bacillarity in year *t* in the logit scale. The color scheme in the table highlights the similarities (diagonally) between *i_t_* and *m*_*t*−1_.

**Table 3:** First differences of the incidence rate in the logit scale.

*t*	2009	2010	2011	2012	2013	2014	2015	2016	2017	2018	2019	2020	2021
*i_t_* − *i*_*t*−1_	−0.03	−0.07	−0.06	−0.07	−0.08	−0.06	−0.03	−0.08	−0.10	−0.08	−0.07	−0.24	−0.04

**Table 4:** Parameters of the incidence model.

Coefficient Name	*β*_0_	*β*_1_	*β*_2_	*β*_3_
Coefficient value	0.14018	1.01082	−0.36095	0.23784
*p*-value	not applicable	8.02 × 10^−11^	4.12 × 10^−5^	0.00118
Residual standard error	0.0174			
Adjusted R-squared	0.9969			

**Table 5:** Parameters of the prevalence model.

Coefficient Name	*γ*_0_	*γ*_1_	*γ*_2_	*γ*_3_
Coefficient value	0.37551	1.05453	−0.29816	0.22458
*p*-value	not applicable	2.51 × 10^−11^	9.18 × 10^−5^	0.000967
Residual standard error	0.01595			
Adjusted R-squared	0.9975			

**Table 6:** Forecast of prevalence of TB with bacterial excretion.

Index/Year	2022	2023	2024	2025	2026	2027
Population screening for TB (conservative scenario) (%)	47.37	58.95	60.00	62.11	63.16	90.00
Prevalence of TB with bacterial excretion (per 100,000)	19.88	20.35	18.33	16.52	14.57	18.28
Population screening for TB (optimistic scenario) (%)	82.11	85.26	86.32	88.42	89.47	90.00
Prevalence of TB with bacterial excretion (per 100,000)	28.66	25.18	20.91	17.48	14.00	10.87

The color scheme highlights the difference in the final indicators.

**Table 7:** TB incidence forecast.

Index/Year	2022	2023	2024	2025	2026	2027
Population screening for TB (conservative scenario) (%)	47.37	58.95	60.00	62.11	63.16	90.00
Incidence of TB (per 100,000)	21.19	23.61	20.64	18.66	16.45	21.15
Population screening for TB (optimistic scenario) (%)	82.11	85.26	86.32	88.42	89.47	90.00
Incidence of TB (per 100,000)	31.22	26.44	21.79	18.31	14.62	11.40

**Table 8:** Predicted and actual values of epidemiological indicators in 2022.

Epidemiological Indicator	Predicted Value in 2022 (95% PI) *	Real Value in 2022
TB incidence (per 100,000)	27.7 (26.4–29.2)	31.1
Prevalence of TB with bacterial excretion (per 100,000)	25.6 (24.5–26.8)	26.6

* PI means “prediction interval”.

## Data Availability

All data generated or analyzed during this study are included in this published article.
